# 1-Benzyl-3-(2-furo­yl)thio­urea

**DOI:** 10.1107/S1600536808006181

**Published:** 2008-03-12

**Authors:** Hiram Pérez, Yvonne Mascarenhas, Osvaldo Estévez-Hernández, Sauli Santos Jr, Julio Duque

**Affiliations:** a Departamento de Química Inorgánica, Facultad de Química, Universidad de la Habana, Habana 10400, Cuba; b Instituto de Física de São Carlos, Universidade de São Paulo, São Carlos, Brazil; c Instituto de Ciencia y Tecnología de Materiales, Universidad de la Habana, Habana 10400, Cuba; d Laboratório de Física, Universidade Federal do Tocantins, CEP 77020-120, Palmas, Tocantins, Brazil

## Abstract

In the title compound, C_13_H_12_N_2_O_2_S, the dihedral angle between the two aromatic ring planes is 87.52 (12)°. The mol­ecule shows an intra­molecular N—H⋯O hydrogen bond. The crystal structure is stabilized by inter­molecular N—H⋯S and C—H⋯O hydrogen bonding.

## Related literature

For general background, see: Estévez-Hernández *et al.* (2007 ▶); Otazo *et al.* (2001 ▶). For related structures, see: Arslan *et al.* (2004 ▶); Khawar Rauf *et al.* (2007 ▶). For the synthesis, see: Otazo *et al.* (2001 ▶).
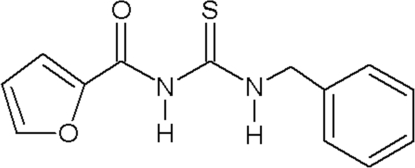


## Experimental

### 

#### Crystal data

C_13_H_12_N_2_O_2_S
*M* *_r_* = 260.31
Tetragonal, 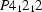

*a* = 9.445 (3) Å
*c* = 27.107 (6) Å
*V* = 2418.2 (12) Å^3^
*Z* = 8
Mo *K*α radiation
μ = 0.26 mm^−1^
*T* = 150 (2) K
0.3 × 0.1 × 0.08 mm

#### Data collection

Nonius KappaCCD diffractometer
Absorption correction: none
12492 measured reflections
2120 independent reflections
1922 reflections with *I* > 2σ(*I*)
*R*_int_ = 0.092

#### Refinement

*R*[*F* ^2^ > 2σ(*F* ^2^)] = 0.035
*wR*(*F* ^2^) = 0.085
*S* = 1.06
2120 reflections
163 parameters
H-atom parameters constrained
Δρ_max_ = 0.17 e Å^−3^
Δρ_min_ = −0.19 e Å^−3^
Absolute structure: Flack (1983 ▶), 802 Friedel pairs
Flack parameter: −0.16 (10)

### 

Data collection: *COLLECT* (Nonius, 2000 ▶); cell refinement: *SCALEPACK* (Otwinowski & Minor, 1997 ▶); data reduction: *DENZO* (Otwinowski & Minor, 1997 ▶) and *SCALEPACK*; program(s) used to solve structure: *SHELXS97* (Sheldrick, 2008 ▶); program(s) used to refine structure: *SHELXL97* (Sheldrick, 2008 ▶); molecular graphics: *ORTEP-3 for Windows* (Farrugia, 1997 ▶); software used to prepare material for publication: *WinGX* (Farrugia, 1999 ▶).

## Supplementary Material

Crystal structure: contains datablocks global, I. DOI: 10.1107/S1600536808006181/xu2401sup1.cif

Structure factors: contains datablocks I. DOI: 10.1107/S1600536808006181/xu2401Isup2.hkl

Additional supplementary materials:  crystallographic information; 3D view; checkCIF report

## Figures and Tables

**Table 1:** Hydrogen-bond geometry (Å, °)

*D*—H⋯*A*	*D*—H	H⋯*A*	*D*⋯*A*	*D*—H⋯*A*
N1—H1⋯O1	0.88	2.00	2.697 (3)	135
N2—H2⋯S1^i^	0.88	2.70	3.578 (2)	174
C7—H7⋯O1^ii^	0.95	2.58	3.423 (3)	148

Symmetry codes: (i) 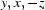; (ii) 
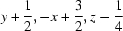
.

**Fig. 1.**
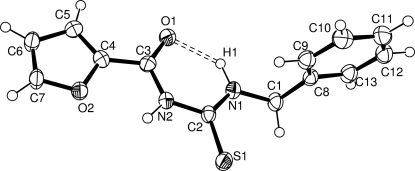
The molecular structure of title compound. Displacement ellipsoids are drawn at the 50% probability level. The intramolecular N—H···O hydrogen bond is shown as a dashed line.

## supplementary crystallographic information

### Comment

Substituted *N*-acylthioureas have been a subject of investigations, due to their ability to form stable metal complexes and as ionophores in potenciometric and amperometric sensors for Cd(II), Hg(II) and Pb(II) (Otazo *et al.*, 2001; Estévez-Hernández *et al.*, 2007). The title compound, (I) (Fig. 1), is another example of our newly synthesized furoylthiourea derivatives, which show outstanding complexation properties.

Compound (I) is a typical N,*N*'-disubstituted thiourea derivative with normal geometric parameters. The C2—S1 and C3—O1 bonds (Table 1) both show the expected double-bond character. The short values of the C2—N1, C2—N2 and C3—N2 bonds indicate partial double bond character.

The dihedral angle between the aromatic rings is 87.52 (12)°, and the angles with the thiourea plane are 86.67 (19)° for the benzene ring and 4.81 (12)° for the furan ring. An intramolecular N–H···O hydrogen bond is present (Table 2), forming a six-membered ring commonly observed in this type of compounds (Arslan *et al.*, 2004; Khawar Rauf *et al.*, 2007). The crystal structure of (I) is stabilized by intermolecular N—H···S and C—H···O hydrogen bonding (Table 2).

### Experimental

The title compound, (I), was synthesized according to a procedure described by Otazo *et al.* (2001) by converting furoyl chloride into furoyl isothiocyanate and then condensing with the appropriate amine. The resulting solid product was crystallized from a dichlorometane-methanol (1:1) mixture yielding X-ray quality single crystals. Elemental analysis for C_13_H_12_N_2_O_2_S found: C 67.73, H 4.75, N 8.23, S 9.34%; calculated: C 67.86, H 4.46, N 8.33, S 9.52%

### Refinement

H atoms were placed in calculated positions with C–H = 0.95 Å (aromatic), N–H = 0.88 Å and C–H = 0.99 Å (methylene), and refined in riding model, *U*_iso_(H) = 1.2*U*_eq_(C,*N*).

### Crystal data

**Table:**

C_13_H_12_N_2_O_2_S	*Z* = 8
*M**_r_* = 260.31	*F*_000_ = 1088
Tetragonal, *P*4_1_2_1_2	*D*_x_ = 1.43 Mg m^−^^3^
Hall symbol: P 4abw 2nw	Melting point: 402.5 K
*a* = 9.445 (3) Å	Mo *K*α radiation λ = 0.71073 Å
*b* = 9.445 (3) Å	Cell parameters from 9761 reflections
*c* = 27.107 (6) Å	θ = 2.9–26.0º
α = 90º	µ = 0.26 mm^−^^1^
β = 90º	*T* = 150 (2) K
γ = 90º	Block, colourless
*V* = 2418.2 (12) Å^3^	0.3 × 0.1 × 0.08 mm

### Data collection

**Table:**

Nonius KappaCCD diffractometer	*R*_int_ = 0.092
ω scans	θ_max_ = 25.0º
Absorption correction: none	θ_min_ = 3.7º
12492 measured reflections	*h* = −11→9
2120 independent reflections	*k* = −8→11
1922 reflections with *I* > 2σ(*I*)	*l* = −32→24

### Refinement

**Table:**

Refinement on *F*^2^	*w* = 1/[σ^2^(*F*_o_^2^) + (0.0402*P*)^2^ + 0.4478*P*] where *P* = (*F*_o_^2^ + 2*F*_c_^2^)/3
Least-squares matrix: full	(Δ/σ)_max_ < 0.001
*R*[*F*^2^ > 2σ(*F*^2^)] = 0.035	Δρ_max_ = 0.17 e Å^−^^3^
*wR*(*F*^2^) = 0.085	Δρ_min_ = −0.19 e Å^−^^3^
*S* = 1.06	Extinction correction: none
2120 reflections	Absolute structure: Flack (1983), 802 Friedel pairs
163 parameters	Flack parameter: −0.16 (10)
H-atom parameters constrained	

### Special details

**Table:**

Geometry. All e.s.d.'s (except the e.s.d. in the dihedral angle between two l.s. planes) are estimated using the full covariance matrix. The cell e.s.d.'s are taken into account individually in the estimation of e.s.d.'s in distances, angles and torsion angles; correlations between e.s.d.'s in cell parameters are only used when they are defined by crystal symmetry. An approximate (isotropic) treatment of cell e.s.d.'s is used for estimating e.s.d.'s involving l.s. planes.

### Fractional atomic coordinates and isotropic or equivalent isotropic displacement parameters (Å^2^)

**Table:**

	*x*	*y*	*z*	*U*_iso_*/*U*_eq_	
C1	0.7270 (2)	0.4402 (2)	0.16180 (8)	0.0311 (6)	
H1A	0.7104	0.3494	0.1791	0.037*	
H1B	0.6351	0.4904	0.1597	0.037*	
C2	0.7259 (2)	0.4740 (2)	0.07225 (7)	0.0251 (5)	
C3	0.8761 (2)	0.3236 (2)	0.01767 (8)	0.0260 (5)	
C4	0.9003 (2)	0.2913 (2)	−0.03448 (8)	0.0270 (5)	
C5	0.9858 (2)	0.1962 (2)	−0.05694 (8)	0.0316 (5)	
H5	1.0486	0.1313	−0.0415	0.038*	
C6	0.9623 (2)	0.2131 (2)	−0.10836 (8)	0.0336 (6)	
H6	1.0069	0.1616	−0.1341	0.04*	
C7	0.8651 (2)	0.3157 (2)	−0.11361 (8)	0.0330 (6)	
H7	0.8296	0.3482	−0.1444	0.04*	
C8	0.8284 (2)	0.5288 (2)	0.19216 (8)	0.0272 (5)	
C9	0.9223 (2)	0.6243 (2)	0.17099 (9)	0.0320 (5)	
H9	0.9287	0.6313	0.1361	0.038*	
C10	1.0065 (3)	0.7093 (3)	0.20046 (10)	0.0389 (6)	
H10	1.0706	0.7739	0.1855	0.047*	
C11	0.9987 (3)	0.7014 (3)	0.25126 (10)	0.0388 (6)	
H11	1.0563	0.7606	0.2713	0.047*	
C12	0.9063 (3)	0.6063 (3)	0.27252 (9)	0.0388 (6)	
H12	0.9001	0.6	0.3074	0.047*	
C13	0.8222 (2)	0.5198 (3)	0.24330 (8)	0.0333 (6)	
H13	0.7598	0.4539	0.2584	0.04*	
O1	0.94160 (17)	0.26029 (17)	0.04999 (6)	0.0314 (4)	
O2	0.82422 (16)	0.36671 (15)	−0.06877 (5)	0.0309 (4)	
N1	0.7763 (2)	0.41033 (19)	0.11201 (6)	0.0275 (4)	
H1	0.8432	0.3465	0.1081	0.033*	
N2	0.77589 (19)	0.42680 (19)	0.02701 (6)	0.0262 (4)	
H2	0.739	0.4679	0.0009	0.031*	
S1	0.60657 (6)	0.60581 (6)	0.072955 (19)	0.03259 (18)	

### Atomic displacement parameters (Å^2^)

**Table:**

	*U*^11^	*U*^22^	*U*^33^	*U*^12^	*U*^13^	*U*^23^
C1	0.0337 (12)	0.0375 (13)	0.0220 (11)	−0.0027 (11)	0.0039 (9)	0.0008 (10)
C2	0.0253 (11)	0.0269 (11)	0.0231 (11)	−0.0052 (9)	0.0015 (9)	−0.0028 (9)
C3	0.0227 (11)	0.0265 (11)	0.0289 (12)	−0.0054 (10)	0.0013 (9)	−0.0005 (9)
C4	0.0289 (12)	0.0274 (12)	0.0248 (11)	−0.0019 (10)	−0.0021 (9)	0.0017 (9)
C5	0.0310 (13)	0.0319 (12)	0.0318 (12)	0.0041 (10)	0.0005 (10)	−0.0019 (10)
C6	0.0396 (14)	0.0353 (13)	0.0260 (12)	0.0033 (11)	0.0041 (10)	−0.0041 (10)
C7	0.0411 (14)	0.0380 (13)	0.0200 (11)	0.0024 (11)	0.0016 (10)	−0.0032 (10)
C8	0.0304 (12)	0.0266 (12)	0.0246 (11)	0.0077 (10)	0.0000 (9)	−0.0018 (9)
C9	0.0354 (13)	0.0324 (12)	0.0283 (12)	0.0043 (11)	0.0009 (10)	0.0006 (11)
C10	0.0381 (14)	0.0358 (13)	0.0427 (15)	−0.0012 (12)	−0.0005 (11)	−0.0003 (11)
C11	0.0399 (14)	0.0381 (13)	0.0385 (14)	0.0034 (12)	−0.0058 (12)	−0.0074 (12)
C12	0.0451 (15)	0.0452 (15)	0.0261 (12)	0.0097 (13)	−0.0045 (11)	−0.0035 (11)
C13	0.0376 (13)	0.0347 (13)	0.0277 (13)	0.0048 (11)	0.0023 (11)	0.0020 (10)
O1	0.0327 (9)	0.0354 (9)	0.0262 (8)	0.0035 (7)	−0.0005 (7)	0.0009 (7)
O2	0.0354 (9)	0.0319 (9)	0.0253 (8)	0.0060 (7)	−0.0011 (7)	−0.0017 (7)
N1	0.0303 (10)	0.0287 (10)	0.0236 (9)	0.0027 (8)	0.0016 (8)	−0.0010 (8)
N2	0.0288 (10)	0.0291 (10)	0.0208 (9)	0.0010 (8)	−0.0008 (8)	−0.0012 (8)
S1	0.0371 (3)	0.0324 (3)	0.0283 (3)	0.0066 (3)	0.0013 (3)	−0.0021 (2)

### Geometric parameters (Å, °)

**Table:**

C1—N1	1.456 (3)	C7—O2	1.363 (2)
C1—C8	1.515 (3)	C7—H7	0.95
C1—H1A	0.99	C8—C9	1.389 (3)
C1—H1B	0.99	C8—C13	1.390 (3)
C2—N1	1.323 (3)	C9—C10	1.384 (3)
C2—N2	1.388 (3)	C9—H9	0.95
C2—S1	1.679 (2)	C10—C11	1.381 (4)
C3—O1	1.228 (3)	C10—H10	0.95
C3—N2	1.382 (3)	C11—C12	1.379 (4)
C3—C4	1.464 (3)	C11—H11	0.95
C4—C5	1.353 (3)	C12—C13	1.387 (4)
C4—O2	1.374 (2)	C12—H12	0.95
C5—C6	1.420 (3)	C13—H13	0.95
C5—H5	0.95	N1—H1	0.88
C6—C7	1.342 (3)	N2—H2	0.88
C6—H6	0.95		
			
N1—C1—C8	114.11 (18)	C9—C8—C1	122.5 (2)
N1—C1—H1A	108.7	C13—C8—C1	118.8 (2)
C8—C1—H1A	108.7	C10—C9—C8	120.3 (2)
N1—C1—H1B	108.7	C10—C9—H9	119.8
C8—C1—H1B	108.7	C8—C9—H9	119.8
H1A—C1—H1B	107.6	C11—C10—C9	120.9 (2)
N1—C2—N2	116.84 (18)	C11—C10—H10	119.6
N1—C2—S1	124.70 (16)	C9—C10—H10	119.6
N2—C2—S1	118.46 (15)	C12—C11—C10	119.1 (2)
O1—C3—N2	123.90 (19)	C12—C11—H11	120.5
O1—C3—C4	120.59 (19)	C10—C11—H11	120.5
N2—C3—C4	115.51 (18)	C11—C12—C13	120.5 (2)
C5—C4—O2	110.61 (18)	C11—C12—H12	119.8
C5—C4—C3	131.8 (2)	C13—C12—H12	119.8
O2—C4—C3	117.63 (18)	C12—C13—C8	120.6 (2)
C4—C5—C6	105.9 (2)	C12—C13—H13	119.7
C4—C5—H5	127.1	C8—C13—H13	119.7
C6—C5—H5	127.1	C7—O2—C4	105.78 (17)
C7—C6—C5	107.0 (2)	C2—N1—C1	123.51 (18)
C7—C6—H6	126.5	C2—N1—H1	118.2
C5—C6—H6	126.5	C1—N1—H1	118.2
C6—C7—O2	110.8 (2)	C3—N2—C2	128.43 (18)
C6—C7—H7	124.6	C3—N2—H2	115.8
O2—C7—H7	124.6	C2—N2—H2	115.8
C9—C8—C13	118.6 (2)		
			
O1—C3—C4—C5	1.4 (4)	C10—C11—C12—C13	0.0 (4)
N2—C3—C4—C5	−178.1 (2)	C11—C12—C13—C8	−0.8 (3)
O1—C3—C4—O2	−179.53 (19)	C9—C8—C13—C12	1.1 (3)
N2—C3—C4—O2	1.0 (3)	C1—C8—C13—C12	−175.5 (2)
O2—C4—C5—C6	0.2 (3)	C6—C7—O2—C4	−0.1 (2)
C3—C4—C5—C6	179.3 (2)	C5—C4—O2—C7	−0.1 (2)
C4—C5—C6—C7	−0.2 (3)	C3—C4—O2—C7	−179.4 (2)
C5—C6—C7—O2	0.2 (3)	N2—C2—N1—C1	−175.32 (19)
N1—C1—C8—C9	27.7 (3)	S1—C2—N1—C1	4.4 (3)
N1—C1—C8—C13	−155.8 (2)	C8—C1—N1—C2	−104.7 (2)
C13—C8—C9—C10	−0.6 (3)	O1—C3—N2—C2	−3.1 (3)
C1—C8—C9—C10	175.9 (2)	C4—C3—N2—C2	176.4 (2)
C8—C9—C10—C11	−0.3 (4)	N1—C2—N2—C3	−2.3 (3)
C9—C10—C11—C12	0.6 (4)	S1—C2—N2—C3	178.05 (17)

### Hydrogen-bond geometry (Å, °)

**Table:**

*D*—H···*A*	*D*—H	H···*A*	*D*···*A*	*D*—H···*A*
N1—H1···O1	0.88	2.00	2.697 (3)	135
N2—H2···S1^i^	0.88	2.70	3.578 (2)	174
C7—H7···O1^ii^	0.95	2.58	3.423 (3)	148

Symmetry codes: (i) *y*, *x*, −*z*; (ii) *y*+1/2, −*x*+3/2, *z*−1/4.
